# How Effective Are DNA Barcodes in the Identification of African Rainforest Trees?

**DOI:** 10.1371/journal.pone.0054921

**Published:** 2013-04-02

**Authors:** Ingrid Parmentier, Jérôme Duminil, Maria Kuzmina, Morgane Philippe, Duncan W. Thomas, David Kenfack, George B. Chuyong, Corinne Cruaud, Olivier J. Hardy

**Affiliations:** 1 Evolutionary Biology and Ecology – Faculté des Sciences, Université Libre de Bruxelles, Brussels, Belgium; 2 Sub-regional Office for Central Africa, Bioversity International, Yaoundé, Cameroon; 3 Canadian Centre for DNA Barcoding, Biodiversity Institute of Ontario, University of Guelph, Guelph, Ontario, Canada; 4 Department of Botany and Plant Pathology, Oregon State University, Corvallis, Oregon, United States of America; 5 Department of Botany, Smithsonian Institution, Washington, D.C., United States of America; 6 Department of Plant and Animal Sciences, University of Buea, Buea, Cameroon; 7 Institut de Génomique – Génoscope, Commissariat à l′énergie atomique et aux énergies alternatives (CEA), Evry, France; University of Guelph, Canada

## Abstract

**Background:**

DNA barcoding of rain forest trees could potentially help biologists identify species and discover new ones. However, DNA barcodes cannot always distinguish between closely related species, and the size and completeness of barcode databases are key parameters for their successful application. We test the ability of *rbcL*, *matK* and *trnH*-*psbA* plastid DNA markers to identify rain forest trees at two sites in Atlantic central Africa under the assumption that a database is exhaustive in terms of species content, but not necessarily in terms of haplotype diversity within species.

**Methodology/Principal Findings:**

We assess the accuracy of identification to species or genus using a genetic distance matrix between samples either based on a global multiple sequence alignment (GD) or on a basic local alignment search tool (BLAST). Where a local database is available (within a 50 ha plot), barcoding was generally reliable for genus identification (95–100% success), but less for species identification (71–88%). Using a single marker, best results for species identification were obtained with *trnH-psbA*. There was a significant decrease of barcoding success in species-rich clades. When the local database was used to identify the genus of trees from another region and did include all genera from the query individuals but not all species, genus identification success decreased to 84–90%. The GD method performed best but a global multiple sequence alignment is not applicable on *trnH-psbA*.

**Conclusions/Significance:**

Barcoding is a useful tool to assign unidentified African rain forest trees to a genus, but identification to a species is less reliable, especially in species-rich clades, even using an exhaustive local database. Combining two markers improves the accuracy of species identification but it would only marginally improve genus identification. Finally, we highlight some limitations of the BLAST algorithm as currently implemented and suggest possible improvements for barcoding applications.

## Introduction

Resources for descriptive taxonomy and biodiversity inventories are much needed to manage the consequences of global change on the world's biodiversity [1]. Unfortunately, these resources are insufficient and they are globally unequally distributed [2]. Moreover, it is likely that taxonomic resources will decline in the future, notably because measures for academic performance, such as the citation index, do not favor basic taxonomic work [3]. The level of accuracy of taxonomic identifications may over- or underestimate the number of species of conservation concern in a community [4]. Taxonomic identification can be difficult, especially for tropical trees: individuals from the same species can vary morphologically according to their age or growing conditions, and closely related species can be morphologically very similar. Reproductive organs such as flowers and fruits are often needed to achieve accurate identification of morphologically similar species, but are often unavailable during field surveys [5]. For instance, in an extensive ecological and molecular survey of nearly 4000 trees belonging to 55 species in the genus *Inga* [6], around 7% of all individuals were incorrectly identified when using morphological characters only. The most common errors involved incorrectly splitting rare morphological variants of common species and incorrectly lumping geographically segregated morphologically similar species. These errors had a measurable impact on ecological analyses. Therefore, developing ancillary methods of taxonomic identification, such as DNA barcoding becomes very important.

DNA barcoding is a molecular and bioinformatics tool used to distinguish between species and to discover new species [7]. It is based on short standardized DNA sequences that should ideally be routinely amplifiable and easily sequenced with “universal” primers (primers anchored in DNA regions highly conserved over large taxonomic range). The utilisation of barcoding for taxonomic identification at species level relies on the expectation that the query species is included in the reference database and that the different species in the database have distinct barcode sequences. In this case, the taxonomic effort can be concentrated on the separation of taxa in the field and in the collection of specimens from which a reference database is constructed. Non-taxonomy specialists can then send samples to a laboratory to get the barcode sequenced, and identify their specimen by matching the sequences to the reference database. Potential barcoding applications are numerous [8]: establishing whether products in international trade belong to protected species, checking the taxonomy of medicinal plants, forensic studies etc.

The mitochondrial marker cytochrome c oxidase I (CO1) has been successfully used for barcoding of some animal groups [9]. It has been more challenging to find a DNA barcode for the identification of a wide range of flowering plants. This is due to several problems including hybridization, lack of sequence polymorphism, frequent introgression between sister species and incomplete lineage sorting. COI barcode is not suitable for plants because relative nucleotide substitution rates of plant mitochondria are lower [10]. Barcoding of plants has focused mostly on plastid DNA (pDNA). Several markers were tested on different plant groups or families and no ideal marker was found (e.g. [5], [11]–[15]). However, a consensus has emerged for using *rbcL* and *matK* plastid genes as standard markers to barcoding land plants [16], although the *trnH-psbA* intergenic region was also promoted as a potential barcode [17].

Assembling DNA barcode libraries is particularly relevant within species-rich natural communities like tropical forests. In two one-ha plots in French Guyana, eight plastid markers were tested and none achieved a rate of correct plant identification greater than 70%, either alone or in combination. However, DNA barcoding was a valuable tool to detect identification errors and for the identification of plants at a juvenile stage [5]. In a 50-ha Forest Dynamics Plot in Panama, barcoding based on three pDNA regions resulted in >98% correct identifications [18]. In a tropical rain forest in Queensland, Australia, in an area with poorly known flora, a DNA-barcoding approach correctly estimated the number of species present in two 0.1 ha plots with almost 90% accuracy. This estimation of species richness at the local scale was obtained from a single survey and without the need of a high level expertise in field identification. It was also obtained in a much shorter time than with the traditional taxonomic methods [19].

The success of a barcoding approach for species identification obviously depends on the exhaustiveness of the database used, because missing species cannot be identified. In the best case, a method could detect that a new species is present [20], but there is a risk that it will assign a tested sample to a wrong species. In addition, the presence of shared haplotypes (i.e. identical DNA sequences) between closely related species is reducing barcoding success and largely explains the limitation of the approach in species-rich communities. An aspect little investigated so far is that while a barcode database might be exhaustive at a local scale in terms of species representation, it is much less likely to be exhaustive in terms of haplotype diversity within species because databases are typically created using a few samples per species. Within species DNA polymorphism is typically low or absent in coding pDNA regions like *rbcL* and *matK* but can be frequent in non-coding regions like *trnH-psbA*.

Nowadays, sequences for African rain forest trees are available on GenBank (http://www.ncbi.nlm.nih.gov/genbank/), but only for a small proportion of the flora: there is no regional-scale barcode database. It is very likely that building an extensive and exhaustive database of barcode sequences for African rain forest trees will take several decades. However, it is possible to construct a local DNA barcode database for an area of special interest. We constructed the first local database for African rain forest trees in the 50 ha forest dynamics plot at Korup in Cameroon. With this database, we test the performances of DNA barcodes in the identification of individual trees to species or genus under the assumption that the database is exhaustive in terms of species content but not necessarily in terms of haplotype diversity within species. We focus on the official standard barcodes *rbcL* and *matK*, and on the noncoding intergenic spacer *trnH-psbA*. There are many analytical methods available for the identification of specimens using barcoding [21], [22]. In this study we compare two methods, one requiring a global multiple alignment and the other based on a basic local alignment search tool (BLAST) which is easy to apply and widely used. Specifically, we address the following questions: (1) How do *rbcL*, *matK* and *trnH-psbA* differ in sequence recovery (first pass comparison)? (2) Are there strong differences in species or genus identification success according to identification methods and markers? (3) Does the gain in information obtained when combining two markers justify the extra cost and labor necessary to sequence a second marker? (4) Does the identification success decrease among closely related species or with intra-species nucleotide polymorphism? (5) Is intra-species polymorphism correlated to local clade richness, as we would expect if related species hybridize, and is it increasing from the local scale to the regional scale? (6) How is barcoding success for species and genus identification affected when a fraction of the species is missing from the reference database, or in the absence of a local database?

## Materials and Methods

### Study sites and sampling

The samples were collected in lowland evergreen forest, in five permanent forest plots where most trees had previously been measured and morphologically identified [23], [24]. A 50 ha plot located in Korup (Cameroon), provided the dataset used for the evaluation of the barcoding resolution at the local scale. Four 1 ha plots located in the Monts de Cristal [24], provided a dataset to study the genus level barcoding success at a regional scale, in absence of a local DNA barcode database. These two areas are particularly interesting for barcoding studies because (i) they have been the focus of intense taxonomic investigation by experienced botanists, (ii) permanent plots are used for ongoing long-term studies and will benefit from our barcoding efforts in future, (iii) the two study areas are located in regions of high tree alpha diversity, where biodiversity monitoring could particularly benefit from a barcoding approach (see Figure 1b in [25]). In both study areas, material for DNA extraction consisted of 5–50 cm^2^ of leaf tissue immediately dried in silica-gel. These samples were included in the African rain forest tree DNA samples collection of the Université Libre de Bruxelles, in the Evolutionary Biology and Ecology laboratory.

The Cameroon plot is located in the Korup National Park (05° 04′ N – 08° 51′ E) and is managed by the Korup Forest Dynamics Plot Programme (KFDP), affiliated with the Center for Tropical Forest Science of the Smithsonian Tropical Research Institute. Climate is equatorial, with mean annual rainfall around 5000 mm and mean annual temperature 30.6°C. Elevation varies between 150 m and 240 m. Around 494 tree species are present in the Korup plot. Due to practical constraints, we sampled only the 272 species represented by more than 50 trees with a diameter at breast height >1 cm according to a 1996 inventory [23], these species representing 97% of the total number of trees in the plot. They belonged to 272 species, 159 genera and 51 families. We collected leaf material from 3 to 4 individuals per species, as along with voucher specimens deposited at the Missouri Botanical Garden (MO), the Herbarium of the Université Libre de Bruxelles (BRLU) and at the KFDP base camp in Mundemba (Cameroon). DNA was extracted for 772 trees among which sequences were obtained for at least one of three barcodes in 725 trees (Table S1).

Samples from Gabon were collected in the Mbé National Park, within four 1 ha plots set up by an international botanical team (two locations: 00° 37′N, 10° 24′E and 00° 28′N, 10° 17′ E, [24]). Annual rainfall is about 3000 mm, mean annual temperature is around 26°C and elevation varies between 250 and 400 m. Only those individuals that belonged to genera present in our Korup dataset were included in this study, i.e. 148 samples belonging to 86 species, 50 genera and 28 families (Table S1). Herbarium voucher specimens were collected for each sample and deposited at BRLU and the National Herbarium of Gabon (LBV). All necessary permits were obtained for the described field studies.

Twenty four of the sampled species were shared between the two sites. In the whole dataset, 18% of the individuals were not identified to a scientifically described species: they belong to morpho-species. These morpho-species represent 17% of the species-level taxa in our dataset in Cameroon and 30% in our dataset in Gabon.

### DNA extraction and sequencing

Protocols for extraction, primer sequences, PCR thermal conditions and sequencing are detailed and referenced in Supporting Information S1. Laboratory work was performed at the Université Libre de Bruxelles (Belgium), in the Biodiversity Institute of Ontario (University of Guelph, Canada), and in the Genoscope (French National Sequencing Center, France). Sequencing was considered successful when bidirectional sequences or two unidirectional sequences obtained in different sequencing runs could be assembled in a reliable contig. We discarded contigs with less than 500 nucleotides for *matK* and *rbcL* and those with less than 180 nucleotides for *trnH-psbA*. We used the ambiguous base coding when two or several base signals were of equivalent strength in the chromatograms. Sequences with a lot of ambiguous bases (>4%) were not included in the dataset.

For *trnH-psbA* we used the trnHf-05 and psbA3f primers. For *rbcL*, two primer pairs were used for different samples in different laboratories: rbcLaF/rbcLaR and rbcL1F/rbcL724R. Amplification of *matK* required the use of two sets of primers (matK1RKim-f/matK3FKim-r and matK390f/matK1326r) to obtain sequences for more than half of the samples. To increase the sequencing success for samples that failed to amplify, we repeated the PCR with the same primer pairs up to four times. Assembling, editing and preliminary alignment of the sequences were done in CodonCode Aligner software (version 3.6.1, CodonCode Corporation).

The sequences were checked in GenBank for possible contaminations. For *matK* and *rbcL,* we also checked the position of the sequence in a neighbour joining tree realised with the PAUP software (v. 4.0b10, [26]) based on what would be expected from the phylogenetic position of the species (APG III phylogeny, [27]). We found several contaminations of Lejeuneaceae species (leafy liverworts) and these sequences were discarded. All sequences have been deposited in the Barcode of Life Data Systems (BOLD, [28]) and will be available on GenBank.

We aligned the sequences for individuals belonging to the same species, and we checked the traces to detect and confirm intra-species sequence polymorphism (note that ambiguous bases were not considered as polymorphism). In the presence of intra-species polymorphism, we carefully compared all reference herbarium vouchers for that species and if we were not certain that the field identification of a sample was correct, this sample was discarded (1% of the samples).

### Testing the barcoding accuracy at the local scale

The local scale analysis was conducted using the Korup dataset. We tested barcoding success when assigning samples to a genus or to a species with our local DNA barcoding reference database under the assumption that the database is exhaustive in terms of species (i.e., the sample to test belongs to a species represented in the database) but not necessarily in terms of within species diversity (i.e., the sample to test may bear an haplotype different from all the conspecific individuals represented in the database). To this end, we excluded the query sample from the database because in a real case study the probability that a query sample comes from an individual already sampled to construct the database is almost zero. In fact, the probability that a query sample bears an haplotype represented in conspecific individuals from the database depends on (i) the haplotypic diversity of the species (number of haplotypes and their relative frequencies at the studied scale), and (ii) the number of conspecific individuals represented in the database. Moreover, keeping a query sample in a database would imply that there is always a sample in the database with a perfect sequence match even for ambiguous or missing bases, which is unrealistic. Consequently, we evaluated the barcoding success only on species represented by at least two samples, so that at least one sample is still in the database when another is the query one.

For the coding genes *rbcL* and *matK*, sequences were aligned using ClustalX implemented in the software Mega4 [29]. We verified and modified the alignment manually where inconsistencies were found, and we translated the sequences into amino-acid sequences to guide the alignment. Sequences were trimmed at both end of the alignment in order to avoid too many missing data at the ends, keeping 705 characters in the alignment for *rbcL* and 1413 for *matK*. We did not align *trnH-psbA* because it was too variable with such a diversity of families. Two methods were used to evaluate barcoding identification success (i.e. the proportion of individuals assigned to the correct species or genus only): the genetic distance between sequence pairs (GD) after a global multiple sequence alignment and the percentage identity (PI) following a basic local alignment search tool (BLAST). As the GD method is based on a global sequence alignment, it was only applied to *rbcL* and *matK*. The PI method was applied to the three markers. Details for the two methods are as follows.

The GD method relied on an analysis of Kimura's 2-parameter (K2P) genetic distance ([30]) matrix among all barcode sequences in the Korup dataset. Analyses were run with the Paup software (v. 4.0b10, [26]). A query sample was matched to the species or genus of the samples from which it was separated by the smallest genetic distance in the matrix (excluding itself). Three situations were considered: (i) the individual was assigned to the correct species or genus because the match corresponded only to samples from its species or genus, (ii) it was assigned to several species or genera including the correct one, (iii) it was assigned to one or several species or genera not including the correct one.

The PI method is similar to the GD method, but the value in the matrix is the minimum dissimilarity between samples, based on the percentage identity, as provided by a BLAST method implemented in Blastclust (version 2.2.23, ftp://ftp.ncbi.nih.gov/blast/executables/release). We used the default settings for the BLAST, except a word count of 20 for *matK*. The commands are given in Supporting Information S1.

An individual was matched to the species or genus of the individuals with which it shared the highest percentage identity in the matrix. We considered only those sequence pairs that had a minimum of 80% percentage identity. We also excluded the sequence pairs with an alignment length inferior to 250 base pairs for *matK* and *rbcL*, and 100 base pairs for *trnH-psbA*.

To calculate the barcoding success for combination of markers, we used the sum of the genetic distances between sequence pairs in the GD method and the sum of the percentage dissimilarity between sequence pairs in the PI method. The significance of differences of barcoding success between markers and/or combination of markers was evaluated with chi-square tests (χ^2^).

We investigated the relationships between the barcoding success and the number of samples per species in the reference database with Spearman correlations. Mean values of the barcoding success were calculated for each species, based on the barcoding success scores with the PI method of all samples belonging to that species. Barcoding success scores were defined as: 1 =  the sample is assigned to the correct species only, 0 =  the sample is assigned to several species including the right one, -1 =  the sample is assigned to one or several species not including the right one. These scores are justified because for a sample that could not be assigned unambiguously to its species, we can consider that a method performs better if it assigns it to multiple species including the correct one rather than to one or several wrong species. With the same method, we tested the relationship between the barcoding success and the species richness of the clade of the query sample in the database. Clade richness was evaluated in two ways: the number of species belonging to the genus of the query sample in the database and the number of species represented by samples sharing at least 99% percentage identity in a BLAST with the query sample. We also tested if the presence of intra-species polymorphism observed in a species (the number of haplotypes) was decreasing the barcoding success for the samples belonging to that species.

Finally, we assessed the influence of the completeness of the database on the species and genus identification success. To this aim, 10%, 20% and 50% of the species were randomly excluded from the reference database. This was done with the PI method and with *rbcL*, using the same query samples as for the analysis with the full database. We did five trials with different random species selections.

### Testing the barcoding accuracy at the regional scale

We evaluated the decrease of accuracy in genus identification when a database from another study area is used, and when this database includes the genus, but not necessarily the species, of the query samples. To this end, we selected the 148 samples in the dataset from Gabon that belonged to a genus represented by at least one individual in the database from the Korup plot in Cameroon. We used the PI method to assign a genus to the specimens in Gabon, using our local database in Cameroon as reference database. The significance of differences of barcoding success between markers and/or combination of markers was evaluated with chi-square tests.

## Results

### Sequencing success

We present here the results obtained for 725 individual trees from the Korup plot in Cameroon (272 species). With a minimum effort (first pass trial: one PCR and sequencing trial per individual), we obtained reliable contigs (contig evaluation is described in the method section) for 77% of the individuals tested for *rbcL*, 71% for *trnH-psbA* and 48% for *matK* (Table 1). Note that these proportions are not perfectly comparable as sequences were obtained in different labs using different protocols. The corresponding percentage of species represented by at least one sample with a good sequence was 94% for *rbcL*, 92% for *trnH-psbA* and 63% for *matK* (Table 1). When repeating the sequencing for individuals that failed previously (up to four times for *matK*), sequencing success over individuals reached 84% for *rbcL,* 80% for *trnH-psbA*, and 64% for *matK*, and in terms of species, 98% for *rbcL*, 97% for *trnH-psbA* and 85% for *matK* (Table 1). The number of good sequences obtained for each species and for each barcode sequence is given in Table S1.

**Table 1:** Sequencing success and intra-specific sequence polymorphism of trees from two African rain forests for *rbcL*, *matK* and *trnH-psbA*.

	*rbcL*	*matK*	*trnH-psbA*
Korup National Park (Cameroon) – 272 sp.			
N ind. tested:	708	620	772
Final sequencing success: N ind. (% ind.)	595 (84%)	397 (64%)	618 (80%)
Final sequencing success: N sp. (% sp.)	266 (98%)	230 (85%)	264 (97%)
Sequencing success at first trial (% ind./% sp.)	77%/94%	48%/63%	71%/92%
N ind. per sp.: mean ± SD (min. – max.)	2.2±0.7 (1–4)	1.7±0.7 (1–4)	2.3±0.8 (1–4)
N sp. with sequences for ≥2 samples	237	139	226
Number of species with several haplotypes:			
- all samples per marker: N sp. (% sp.)	5 (2%)^a^	7 (5%)^a^	42 (19%)^b^
- 219 samples from 102 sp. with seq. for the 3 barcodes and ≥2 ind./sp.: N sp. (% sp.)	3 (3%)^a^	4 (5%)^a^	28 (27%)^b^
Korup National Park (Cameroon) and Monts de Cristal (Gabon) – 24 shared sp.			
N sp. with good seq. in both forests	23	12	13
Species with several haplotypes: N sp. (% sp.)	6 (26%)^a^	1 (8%)^a^	9 (70%)^b^

sp.: species, ind.: individuals, seq.: sequences, N: number.

Shared superscript letters indicate markers that do not differ significantly in the proportion of species with several haplotypes (χ^2^ tests).

### Barcoding accuracy at the local scale

The performances of the three markers with the two barcoding identification methods within a 50 ha plot in Cameroon are presented in Table 2. Two series of results are presented: one with all the samples available for each marker, and the other for the samples successfully sequenced at all three markers where results are given for each marker or combination of markers. We use the latter to for our detailed comparison between markers below, but the trends are globally similar when we consider all samples per marker.

**Table 2:** Barcoding success of African rain forest trees at a local scale for species identification (a) and genus identification (b).

(a) Species identification	Correct (%)		Multiple/Wrong (%)		Query samples			Barcoding database		
	GD	PI	GD	PI	N. ind.	N. sp.	N. ge.	N. ind.	N. sp.	N. ge.
All samples with good quality sequences										
*rbcL*	71.9^a^	71.2^a^	28.0/0.2	25.7/3.2	565	237	88	594	266	161
*matK*	76.5^ a^	75.5^a^	22.2/1.3	17.6/6.9	306	139	100	396	230	145
*trnH-psbA*	/	84.3^b^	/	12.6/3.1	579	226	144	617	264	157
Samples with good quality sequences available for the 3 markers										
*rbcL*	72.6^a^	71.7^a^	27.4/0	26.9/1.4	219	102	72	325	211	141
*matK*	74.9^a^	74.9^ab^	22.8/2.3	15.1/10	219	102	72	325	211	141
*trnH-psbA*	/	80.8^bc^	/	14.6/4.6	219	102	72	325	211	141
*rbcL* + *matK*	83.1^b^	79.5^abc^	15.1/1.8	16.4/4.1	219	102	72	325	211	141
*trnH-psbA + rbcL*	/	85.6^bcd^	/	9.6/4.6	219	102	72	325	211	141
*trnH-psbA + matK*	/	85.8^cd^	/	7.8/6.4	219	102	72	325	211	141
*rbcL + matK + trnH-psbA*	/	87.7^d^	/	5/7.3	219	102	72	325	211	141

(b) Genus identification	Correct (%)		Multiple/Wrong (%)		Query samples			Barcoding database		
	GD	PI	GD	PI	N. ind.	N. sp.	N. ge.	N. ind.	N. sp.	N. ge.
All samples with good quality sequences										
*rbcL*	96.9^a^	96.8^b^	3.1/0	2.7/0.5	554	233	142	583	262	158
*matK*	97.3^a^	93.8^a^	2.3/0.3	2.3/1.6	299	136	97	388	226	143
*trnH-psbA*	/	98.6^c^	/	0.2/1.2	572	226	143	609	261	154
Samples with good quality sequences available for the 3 markers										
*rbcL*	99.1^ab^	98.1^bc^	0.9/0	0.9/0.9	215	100	70	319	205	135
*matK*	96.7^a^	94.4^a^	3.3/0	2.3/3.3	215	100	70	319	205	135
*trnH-psbA*	/	97.7^abc^	/	0/2.3	215	100	70	319	205	135
*rbcL* + *matK*	100^b^	95.8^ab^	0/0	1.4/2.8	215	100	70	319	205	135
*trnH-psbA + rbcL*	/	100^d^	/	0/0	215	100	70	319	205	135
*trnH-psbA + matK*	/	99.1^cd^	/	0/0.9	215	100	70	319	205	135
*rbcL + matK + trnH-psbA*	/	99.2^cd^	/	0/0.8	215	100	70	319	205	135

Shared superscript letters indicate which pDNA regions or combinations of pDNA regions do not differ significantly in their barcoding success (% correct identification, χ^2^ tests).

Two methods were used to evaluate barcoding identification success: the minimum genetic distance between sequence pairs (GD) and the maximal percentage identity in a basic local alignment search tool (PI). Correct identification  =  the individual is assigned to the correct species or genus only, multiple identification  =  the individual is assigned to several species or genera including the right one, wrong identification  =  the individual is assigned to one or several species or genera not including the right one. Only those individuals with at least one other individual of their species in the database were tested against databases containing all the available samples, except the query individual. N.: number, sp.: species, ge.: genera, ind.: individual. Note that six morpho-species belonging to unknown genera were excluded from the reference databases for genus-level identification but were kept for species-level identification.

With the GD method, best results were obtained with the *rbcL*+*matK* combination, with 83% successful species identifications, versus 73% and 75% for *rbcL* or *matK* alone. At the genus level, the added value of combining *matK* to *rbcL* was marginal because *rbcL* alone was successful for 99% of the samples.

In this study, the three markers could only be compared with the PI method because the GD method requires a global multiple sequence alignment which is not possible for *trnH-psbA*. Species identification was successful for 81%, 72% and 75% of the samples for *trnH-psbA, rbcL* and *matK*, respectively. Combining *trnH-psbA* with *rbcL* or *matK* provided 86% success, compared to 80% with the *rbcL*+*matK* combination, but the difference is not statistically significant. Combining the three markers further increased species identification success to 88%. At the genus level, *rbcL* and *trnH-psbA* each provided 98% success and *matK* 94%. Combining *trnH-psbA* with *rbcL* or *matK* provided 99% to 100% success, values significantly higher than the 96% success for the *rbcL*+ *matK* combination, and not significantly different from the 99% success obtained when combining the three markers.

The two methods and the markers also differed in the degree of “wrong” identifications, i.e. when the method matched the query sample to one or several species not including the correct one (see Table 2). The percentages of wrong species identifications were always lower with the GD method (between 0 and 2.3%) than with the PI method (between 1.4 and 10%).

The success rate for species identification was not affected by the number of samples per species, but it was strongly influenced by the clade richness of the query sample (Spearman correlations −0.364 to −0.627, P>0.001, Table 3). The correlation between the number of haplotypes of a species within the reference database and the mean barcoding success of the samples belonging to that species was significant for *matK* (Spearman correlation −0.178, P = 0.04) but not for the other markers (Table 3).

**Table 3:** Determinants of the barcoding success of African rain forest trees: Spearman's correlation coefficients between the proportion of individuals correctly identified at the species level and the number of individuals (N. indiv), number of haplotypes (N. haplotypes), or clade richness (Clade R. genus, Clade R. 99% PI) per species in the database.

Mean Barcoding success	N. indiv	N. haplotypes	Clade R. genus	Clade R. PI
*matK*	−0.103 NS	−0.178*	−0.431***	−0.477***
*rbcL*	−0.114 NS	0.015 NS	−0.485***	−0.417***
*trnH-psbA*	0.054 NS	−0.123 NS	−0.364***	−0.627***

P-values of tests: * P≤0.05, *** P<0.001, NS non significant (P>0.05.) The barcoding success is calculated for each species as the mean barcoding success of all individuals belonging to that species (1: assigned to the correct species only, 0: assigned to several species including the correct one, −1: assigned to one or several species not including the right species). Clade richness is either measured as the number of species in the database belonging to the same genus as the query individual (Clade R. genus), or as the number of species in the database that have samples with a percentage identity in a BLAST ≥99% with the query sample (Clade R. PI).

Unsurprisingly, the success rate for species and genus identification decreased when the reference database did not include all the species of the query samples. For *rbcL*, the species identification success dropped from 71.2% with the full database, to 67.1±1.6 %, 61.7±1.7 % and 42.5±2.4 % using a database with 10, 20 or 50% missing species, respectively. The genus identification success dropped from 96.8% to 92.0±1.3 %, 87.5±0.7 % and 71.0±2.8 % using a database with 10, 20 or 50% missing species, respectively.

### Barcoding accuracy at the regional scale

At the regional scale, we tested the effectiveness of the PI method for genus identification when blasting the 148 trees from the dataset in Gabon belonging to genera present in our local database of barcode sequences from Cameroon. Here, the genera, but not all species, of the query samples were present in the reference database. Identification success reached 84% with *rbcL*, 88% with *trnH-psbA* and 90% with *matK* but these differences are non significant (Table 4; χ^2^ tests, P>0.05). The percentages of wrong genus identifications varied between 8 and 12%. Combining the markers did not improve the success rate compared to using *matK* alone.

**Table 4:** Barcoding success of African rain forest trees at the regional scale for genus identification using the PI method.

	Correct (%)	Multiple/Wrong (%)	N	
All samples				
*rbcL*	83.9	6.3/9.8	143	
*matK*	85.0	10.0/5.0	80	
*trnH-psbA*	88.6	0.0/11.4	88	
Samples available for the 3 barcodes				
*rbcL*	84.3	5.9/9.8	51	
*matK*	90.2	2.0/7.8	51	
*trnH-psbA*	88.2	0.0/11.8	51	
*rbcL + matK*	86.3	3.9/9.8	51	
*rbcL + trnH-psbA*	90.2	0.0/9.8	51	
*matK + trnH-psbA*	90.2	0.0/9.8	51	
*rbcL + matK + trnH-psbA*	90.2	0.0/9.8	51	

All χ^2^ tests for differences in barcoding success (% correct identification) among markers or combinations of markers were non-significant.

These results are obtained from an analysis of the highest percentage identity resulting from a BLAST of DNA sequences from samples in Gabon on a local reference database from Cameroon. The reference database contains at least one individual of the genus of the individuals from Gabon, but not always one individual of their species. Correct  =  the percentage of samples assigned to the correct genus only, multiple  =  the percentage of samples assigned to several genera including the right one, wrong  =  the percentage of samples assigned to one or several genera not including the right one. N: number of query samples tested.

### Intra-species polymorphism

At the local scale, the percentage of species showing intra-species polymorphism differed significantly between *trnH-psbA* and the two other markers (χ^2^ tests, P<0.001): 2% with *rbcL*, 5% with *matK* and 19% with *trnH-psbA* (Table 1, Table S1). To test the hypothesis that intra-species polymorphism increases through hybridization between closely related species, we tested the correlation between intra-species polymorphism and the species richness of the genus to which a species belongs (based on the list of the 494 tree species present in the 50 ha plot), and it was not significant (Spearman correlation coefficient  =  −0.07, P>0.05).

Compared to the local scale, the percentage of species showing polymorphism strongly increased at the regional scale, when we added the individuals from Gabon to those from Cameroon (Table 1). This increase mostly corresponds to differences between individuals from Gabon and Cameroon. However, for several species, only one of the individuals from one country was different from those in the other country (Table S1).

## Discussion

### Sequence recovery and practical considerations

Among the three barcode markers, *matK* required much more effort to retrieve the sequences than *rbcL* and *trnH-psbA*. Because we used different protocols in different labs and for different makers, we were not able to make a statistical analysis to measure the lab effect and the marker effect, and this result is therefore only indicative. However, a lower amplification and sequencing success of *matK* has been reported in several other studies (i.e. 42 % of the species [12] or around 70% [5], [18], [31], [32]). In contrast, the CBoL Plant Working Group [16], on a dataset of 367 angiosperms samples, reported that 84% of the Angiosperm species were successfully amplified and sequenced using a single *matK* primer pair. We used the same primer pair on our dataset, and on the first trial we obtained reliable sequences for only 63% of the species. We needed to use two different pairs of primers and up to four trials to get sequences for 85% of the species. Fazekas *et* *al.* [33] reported to have obtained *matK* sequences for 91% of the species they tested, but they used up to 10 primer pairs. Recently, however, new primers for *matK* were designed that might improve the sequencing success [34].

If an alignment of all sequences is needed in the method used for the assignment (e.g. GD method), *rcbL* is certainly the easiest choice. Indeed, when mixing samples from a large number of families, *matK* is more difficult to align than *rbcL*, and it was impossible, given its high level of inter-species polymorphism, to align *trnH-psbA* sequences in one unambiguous alignment. This is a common difficulty with non-coding sequences [35]. Another advantage when aligning *rbcL* is that there are no gaps and only one alignment is possible, while *matK* often contains indels so that several equally acceptable alignments are possible. Using the coding genes (*rbcL* and *matK*) provides an additional assessment of sequence quality, because these sequences should match the reading frame, which reduces the risk of error due to missing bases or duplication, and allows the detection of nuclear copies of plastid fragments that are sometimes sequenced together with pDNA [36].

### Barcoding identification accuracy

At a local scale, best results for species identification using only one marker were obtained with *trnH-psbA* (81%). For genus identification, *rbcL* and *trnH-psbA* gave comparable results (98%) and were significantly better than *matK*. However, for genus identification at a regional scale (Table 4), *matK* performed best (90%) and *rbcL* was the least effective (84%), but differences are not statistically significant. Hence, results are highly context dependent and it seems difficult to draw general conclusions on the relative performances of the three barcodes.

Combining two markers improved the barcoding success at the species level by 7%. However, for genus identification, *rbcL* alone provided 99% successful identifications at a local scale and combining it to *matK* or *trnH-psbA* is therefore much less relevant. Note that both for species and for genus identification, the combination of *trnH-psbA* with one of the two other markers was more successful than the *rbcL+matK* combination.

Our results are comparable with other DNA barcoding studies of tropical trees conducted at a community scale. For example, in French Guiana, Gonzalez *et al.* [5] obtained a rate of species identification ranging from 60% to 75% for the three markers we used. By contrast, in Panama and in Puerto Rico, Kress *et al.* [18], [31] reported much higher rates (reaching 94–100% in Puerto Rico and 75–99% in Panama), the lowest values being obtained for *rbcL*. These differences might partly be due to the different floristic contexts, the reported identification rates decreasing with increasing mean number of species per genus (1.3 in Puerto Rico, 1.6 in Panama, 1.7 in Cameroon, 1.8 in French Guiana). However, differences in the methods applied might also affect the results. For example, in their BLAST approach, Kress *et* *al.* [18], [31] did not exclude the query sample from the database tested and considered the highest Bit-Score rather than the highest Percentage Identity. Applying this approach to our dataset using all available samples per pDNA sequence, we obtain 75.3%, 81.1% and 92.1% species identification success for *rbcL*, *matK* and *trnH-psbA*, respectively, which is 4% to 8% better than the values reported in Table 2a. However, we believe that these results are too optimistic. Indeed, even if two sequences from different species are identical except for a few unresolved bases or a slight difference in length, the Bit-Score will be higher between a sequence and itself than between the two sequences (see Table 5 for an example). This will overestimate the actual identification success rate. The study by Gonzalez *et al.* [5] used, among others, a BLAST-based clustering approach which likely provides a more stringent criterion than our approach and might also explain the lower identification rates reported.

**Table 5:** Impact of sequence length differences, ambiguous bases or missing data on K2P distance and the output of the BLAST algorithm (Percentage Identity and Bit-Score).

Query sample	Subject sample	K2P	BLAST output				
			PI	alig. length	Mismatches	Bit-Score	% Bit-Score max.
seq_ok	seq_ok	0	100	368	0	729	100
seq_ok	seq_N	0	99	368	3	712	98
seq_ok	seq_Y	0	99	368	3	718	98
seq_ok	seq_short	0	100	354	0	702	96

seq_ok is a 368 bp long sequence without missing data or ambiguous bases. It is compared to that same sequence with slight modifications representative of the limits of sequencing techniques: seq_N has three “N” within the sequence (internal missing data), seq_Y has three “C” or “T” bases replaced by a “Y” (ambiguous bases), seq_short is 14 bp shorter (missing data at each end). K2P: K2P distance obtained with the PAUP software. PI (Percentage Identity) and Bit-Score result from a BLAST analysis obtained with the BLASTCLUST software. % Bit-Score max. is the percentage of the Bit-Score obtained compared to the maximum Bit-Score (when seq_ok is blasted on itself).

When comparing the power of the two methods we applied to identify the correct species using *rbcL*, *matK* or their combination, the GD method always performed better or as well as the PI method. Moreover, the risk of misidentification was always lower with the GD method (up to 2%) while it could reach ca. 10% with the PI method. In fact, as illustrated in Table 5, the efficiency of the PI method was reduced in the presence of ambiguous bases because potentially identical bases are treated as different in the calculation of the percentages identity provided by the BLAST algorithm, while they are considered as identical in the calculation of the K2P distance (GD method). This difference substantially increases the percentage of wrong identifications with the PI method. If a query sequence is identical to the sequences of say two species in the database, an ambiguous base occurring in the sequence of the correct species will cause the PI method to match the query sequence to the wrong species while the GD method will conclude that there are multiple possible species assignments (i.e. that there are multiple species with equal genetic distance to the query). This problem with the PI method could probably be solved through a modification of the BLAST algorithm. Using the Bit-Score rather than the PI as criterion for species identification worsens the problem because, as stated above, the Bit-Score also depends on sequence length (Table 5). Further improvements of identification success could possibly be obtained with new methods, like the one designed by Little [21], incorporating taxon hierarchy and within-taxon variability, or character-based approaches (e.g. [37]). Nevertheless, simulation results involving closely related species report only a marginally higher performance of diagnostic-based methods over distance or BLAST methods [38]. The likely reason is that identification success is mostly limited by the occurrence of shared haplotypes between species, a limit that no method can circumvent.

For all barcode markers, there was a significant decrease in successful species identification when the clade richness increased (Table 3). This is unfortunate because it is precisely for closely related species difficult to identify in the field without fertile specimens (e. g. in the genera *Beilschmiedia, Cola, Rinorea, Trichoscypha.*..) that barcoding identification would be most useful. This lower performance of barcoding in species-rich clades might in part be explained by an under or over-estimation of the diversity in the field. Indeed, in our dataset, we had 17% morpho-species that have not yet been matched to a scientifically described taxon. Some of these morpho-species might belong to species complexes with little morphological differences between individuals that could have been grouped under the same name in our dataset. On the other hand, it is possible that within some genera, morpho-species have been created that do not represent real species, but rather variants of the same species. However, the lower performance of barcoding in species-rich clades might also reflect a limitation of DNA barcoding based on plastid markers. There are many examples of poor resolution of the barcode sequences for sister species (e.g. in *Crocus* [13] and *Quercus* [39], but sister species were well differentiated in *Acacia* [40]). The resolution of the barcodes in distinguishing sister species probably differs according to the evolutionary history of the group concerned. Lahaye *et al.* [41] estimated the barcoding gap for *matK* on a large number of samples from biodiversity hotspots in Costa Rica and in southern Africa. The distributions and means of intra-specific differences were lower than for interspecific divergences. They did not however find any large barcoding gap.

The use of DNA sequences as barcodes to discriminate between species is based in part on the assumption that species bear unique barcode haplotypes. But large percentages of species were found to share haplotypes in several barcoding studies (e.g. [33], [41]). Plastid haplotype sharing might reflect three distinct phenomena: (i) gene exchange caused by hybridization and/or polyploidy; (ii) incomplete sorting of ancestral polymorphisms or insufficient rate of molecular evolution; and (iii) imperfect species definition and taxonomy [35]. Under the hybridization hypothesis, we would expect higher intra-specific polymorphism within species-rich genera, which was not the case in this study. The frequent absence of polymorphism within genera rather indicates a low rate of molecular evolution. As was stated by Casiraghi *et al.* [42], the biological meaning of the molecular entities identified with the barcode cannot be directly derived unless we have clearly and unequivocally linked a species to the variability pattern of a single DNA barcoding marker. Plant DNA barcoding has mostly focussed on plastid genes. However, there is evidence of plastid captures between closely related species, including in the African flora (e.g. [43], [44]). Hence, to be effective, DNA barcodes within genera where plastid capture can occur between species must rely on nuclear genes.

Sequences availability in the database is a major limiting factor of DNA barcoding. Databases like BOLD or GenBank are general databases (not local), and will probably never be complete. These databases will however be used for barcoding unidentified specimens. In a real case study, it is not unrealistic that a non negligible proportion of samples will belong to species absent from the reference database, which will increase the rates of unassigned samples and of wrong identifications. Our results indicate that it is still reasonable to use a reference database including only 80% of the species present in the study area for a genus level identification (87% identification success with *rbcL* alone), which can be very useful in highly diverse forests. This is confirmed by our study conducted at the regional scale (without local database) where 90% correct genus identification was achieved using samples from genera, but not necessarily species, included in the database.

### Intra-species polymorphism

Intra-species polymorphism with the *rbcL* and *matK* markers was generally limited to substitution of one or a few bases, while *trnH-psbA* sequences were often very variable between individuals. This higher intra-specific variation level of *trnH-psbA* has been reported in several other studies (i.e. [18], [19]). Consequently, *rbcL* and *matK* sequences were more effective at detecting field misidentifications. In our study, when *rbcL* and *matK* sequences from individuals attributed to the same species were divergent, we carefully checked the reference herbarium vouchers and in most cases it was a problem of incorrect taxonomic identification.

At the regional scale, the percentages of species showing intra-specific polymorphism considerably increased for all three markers. This could result from problems of taxonomic identifications as different botanical teams worked in the two study sites. However, in several cases, the intra-species polymorphism was not distinguishing samples from Gabon and from Cameroon. Moreover, taxonomic identification errors were limited in our dataset because we checked the herbarium samples carefully. We have good reasons to think that even well-delimited species can be polymorphic at *rbcL* and/or *matK* in the African flora. This was observed for example in *Santiria trimera*, a complex of species where *rbcL* polymorphism was detected even within a well-defined morphotype in Gabon [36]. Further population genetics studies for this morphotype demonstrated that it forms a well-defined gene pool matching the biological species concept (Koffi *et al.*, unpublished). An increase of plastid genetic diversity from the local scale to the regional scale was also observed in other studies (i.e. [43], [44], [45]). Therefore, a few percent of species showing *rbcL* or *matK* polymorphism at the local scale does not necessarily reflect taxonomic identification problems.

Classical taxonomic studies screen numerous individuals from multiple localities across the range of a given species to distinguish variation within a species from variation between species, in order to identify those characters that are uniquely shared among all members of that species [7]. Similarly, a reference database for the barcoding of African rain forest trees should include sufficient sequences for each species distributed over all its distribution range in order to be representative of its intra-specific variability.

## Supporting Information

Supporting Information S1 **Protocols for extraction, primer sequences, PCR thermal conditions and sequencing and BLAST commands.**
(DOC)

Table S1 **List of the herbarium samples, BOLD ID, GenBank accession numbers and summary of the sequencing success and intra-specific polymorphism.**
(XLS)
