# Supplementary material for: How Effective Are DNA Barcodes in the Identification of African Rainforest Trees?
Source: PLoS One. 2013 Apr 2;8(4):e54921. doi: 10.1371/journal.pone.0054921 (PMC3615068; doi:10.1371/journal.pone.0054921)
Supplement: Supporting Information S1 — Protocols for extraction, primer sequences, PCR thermal conditions and sequencing and BLAST commands. (DOC) [file pone.0054921.s001.doc]

**Supporting Information S1.** Protocols for extraction, primer sequences, PCR thermal conditions and sequencing, and BLAST commands.

Laboratory work was done at the Université Libre de Bruxelles (ULB, Belgium), in the Biodiversity Institute of Ontario (University of Guelph, Canada) and in the Genoscope (French National Sequencing Center, France). (a) Primer sequences, (b) Protocols used at the Biodiversity Institute of Ontario (University of Guelph, Canada), (c) Protocols used at the Université Libre de Bruxelles, (d) Protocols used at the Genoscope.

**(a) Primer sequences**

***rbcL* primers**

rbcLa-F ATGTCACCACAAACAGAGACTAAAGC Levin, 2003

rbcLa-R GTAAAATCAAGTCCACCRCG Kress *et al.* 2009

rbcL1f ATGTCACCACAAACAGAAAC Fay *et al.* 1997

rbcL724r TCGCATGTACCCTGCAGTAGC Olmstead *et al.* 1992

***matK* primers**

MatK-1RKIM-f ACCCAGTCCATCTGGAAATCTTGGTTC Ki-Joong Kim, Unpublished

MatK-3FKIM-r CGTACAGTACTTTTGTGTTTACGAG Ki-Joong Kim, Unpublished

MatK_390f CGATCTATTCATTCAATATTTC Cuenoud *et al.* 2002

MatK_1326r TCTAGCACACGAAAGTCGAAGT Cuenoud *et al.* 2002

***trnH-psbA primers***

psbA3’f GTTATGCATGAACGTAATGCTC Sang et al. 1997

trnHf-05 (reverse) CGCGCATGGTGGATTCACAATCC Tate & Simpson, 2003

**References for the primers**

Cuenoud P, Savolainen V, Chatrou LW, *et al*. (2002) Molecular phylogenetics of Caryophyllales based on nuclear 18S rDNA and plastid *rbc*L, *atp*B, and *mat*K DNA sequences. *American Journal of Botany*, 89: 132-144.

Fay M. F., Swensen S. M., Chase M. W. (1997). Taxonomic Affinities of *Medusagyne oppositifolia* (Medusagynaceae). *Kew Bulletin*, 52(1): 111-120.

Kress, W. J., Erickson, D. L., Jones, F. A., Swenson, N. G. *et al.* (2009). Plant DNA barcodes and a community phylogeny of a tropical forest dynamics plot in Panama. *Proceedings of National Academy of Sciences USA*, 106 (44): 18621-18626.

Levin RA, Wagner WL, Hoch PC, *et al*. (2003) Family-level relationships of Onagraceae based on chloroplast *rbc*L and *ndh*F data. American Journal of Botany, vol 90:107-115 (modified from Soltis P *et al*. (1992) *Proceedings of National Academy of Sciences USA*, 89: 449-451).

Olmstead. R. G., Michaels, H. J., Scott, K. M. & Palmer, J. D. (1992). Monophyly of the Asteridae and identification of their major lineages inferred from DNA sequences of *rbcL*. *Ann. Missouri Bot. Gard.*, 79: 249 - 265.

Sang T, Crawford DJ, Stuessy TF (1997) Chloroplast DNA phylogeny, reticulate evolution and biogeography of Paeonia (Paeoniaceae). *American Journal of Botany* 84: 1120–1136.

Tate JA, Simpson BB (2003) Paraphyly of Tarasa (Malvaceae) and diverse origins of the polyploid species. *Systematic Botany* 28: 723–737.

**(b) Protocols for extraction, PCR thermal conditions and sequencing used at the Biodiversity Institute of Ontario (University of Guelph, Canada)**

http://www.dnabarcoding.ca/

The detailed protocols are available online:

<http://www.ccdb.ca/CCDB_DOCS/CCDB_DNA_Extraction-Plants.pdf>
<http://www.ccdb.ca/CCDB_DOCS/CCDB_Amplification-Plants.pdf>

And also in Fazekas et al. (2012) DNA barcoding methods for land plants *In*: W. John Kress and David L. Erickson (eds.), *DNA Barcodes: Methods and Protocols*, Methods in Molecular Biology, vol. 858: 223-252, DOI 10.1007/978-1-61779-591-6_11, Springer Science

**DNA extraction:**

1. Prior to processing, the plant boxes were centrifuged at 1500 g for 2 min.
2. One stainless steel bead was added to each tube with dry tissue (silica gel) and covered with fresh strip caps. The boxes with removed lids were inserted into TissueLyser (Qiagen) adapters and shaked at 28 Hz for 30 sec, then rotated and shaked one more time, then centrifuged at 1500 g for 2 min.
3. The strip caps were opened very carefully by pulling each cap away from the well to avoid cross-contamination. 250-350 μL of 2×CTAB was added to each tube and covered with new strip caps.
4. The boxes were gently inverted once and centrifuged at 1500 g for 1 min., then incubated at 65ºC for 1.5 hours.
5. 50 μL of lysate from each sample was transferred into a 96-well Eppendorf plate.
6. 100 μL of Plant Binding Buffer (PBB) was added to each sample using multichannel pipette or Liquidator 96 (Mettler Toledo), gently mixed with pipette tips 5-10 times and incubated for 5 min at RT.
7. 150 μL of lysate was transferred from the wells of the microplate into the wells of the GF plate (PALL1 or PALL2) placed on top of a square-well block using multichannel pipette. The plate was sealed with self-adhering foil and centrifuged at 5000 g for 5 min.
8. 750 μl of Wash Buffer (WB) was added to each well of the GF plate, sealed with a new self-adhering foil and centrifuged at 5000 for 5 min.
9. The GF plate with removed adhering foil was placed on top of the inverted sterile lid from a tip box and incubated at 56 °C for 30 min.
10. The GF plate was placed on top of the collection microplate and DNA was eluted with 50 – 60 μL of ddH20 (prewarmed to 56°C), incubated at room temperature for 1 min, and sealed.
11. The assembled plates were placed on a clean square-well block to prevent cracking of the collection plate and centrifuge at 5000 g for 5 min to collect the DNA eluate. The GF plate was removed and discarded.

Working solutions for DNA extraction:


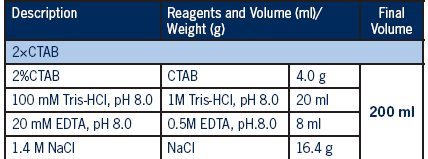


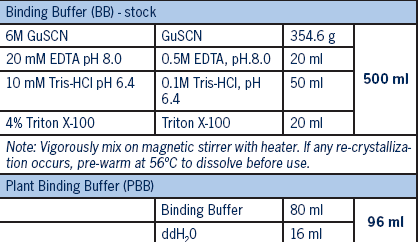


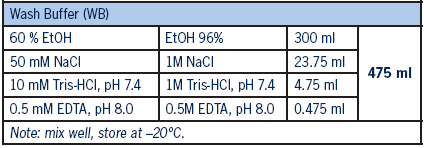


**PCR amplification of the fragments:**

PCR mix for rbcL :


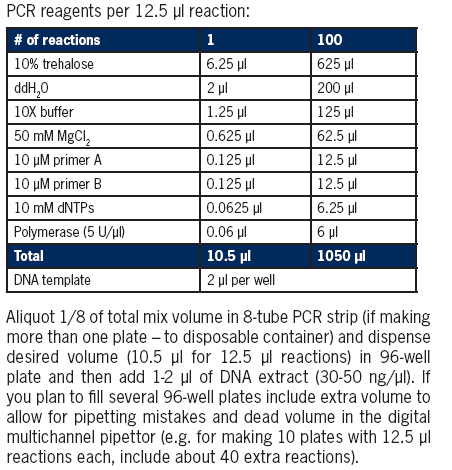


PCR mix for *matK* :


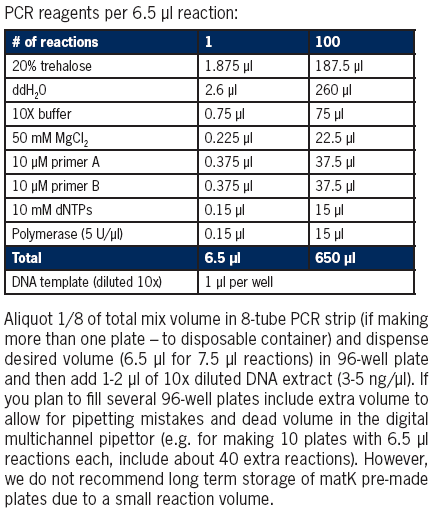


PCR mix for *trn*H*-psb*A


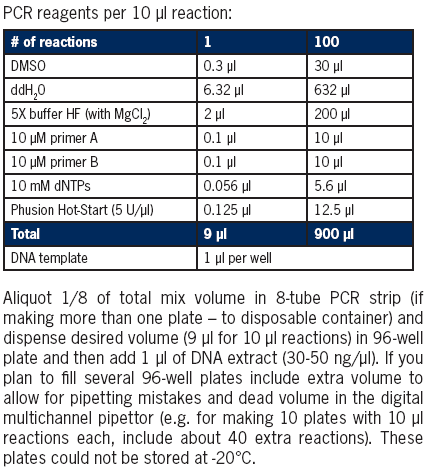


PCR thermocycler programs :


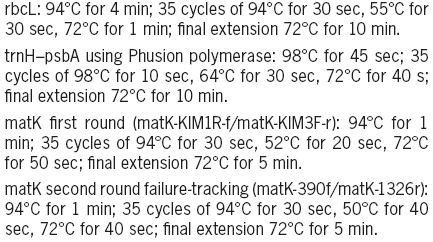


**Cycle Sequencing**

1. The PCR products were diluted:

(a) For *rbc*L and *trn*H– *psb*A: one part of PCR product/two parts of water.

(b) For *mat*K: one part of PCR product/nine parts of water.

2. The plate was covered with plastic seal, and spin at 1,000 × *g* force for

1 min.

3. The sequencing reagents were defrosted (Table 5) at room temperature.

4. The sequencing mix was prepared adding components in the order listed

in Table 5 .

5. 9.0 μL of sequencing mix was dispensed into each well of 96-well plate and 2 μL of diluted PCR product was added to each well.

7. The heat-seal cover was placed over the top of the 96-well plate and heat and roller applied to close the plate tightly.

8. The plate was spin using centrifuge at 1,000 × *g* for 1 min. and placed into the thermocycler block. The following program was applied: 96°C for 2 min; 30 cycles of 96°C for 30 s, 55°C for 15 s, 60°C for 4 min; hold at 4°C.

10. After cycle sequencing reaction was completed, the plate was kept in a dark box at 4°C to avoid degradation of BigDye™.


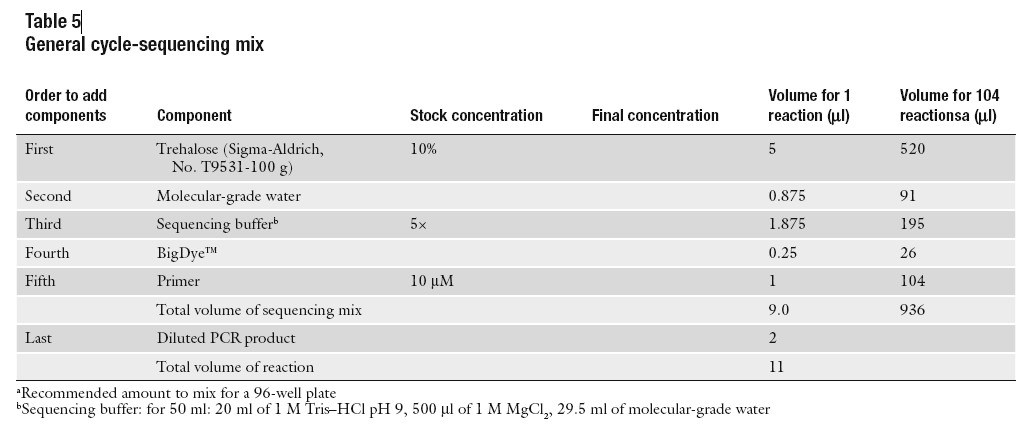


**Cycle Sequencing Cleanup and Processing for an ABI 3730xl Capillary Sequencer**

1. Dry Sephadex G-50 (Sigma-Aldrich, Cat. No. G5080-500 g) was measured with the MultiScreen Column Loader (Millipore, Cat. No. MACL09645) into the Acroprep 96 Filter plate with 0.45 μM GHP membrane (PALL, Cat. No. PN5030).

2. Each well was hydrated with 300 μL of molecular-grade water using a pipette and left overnight at 4°C before use.

4. The Sephadex plate was assembled with the collection plate, secure with two rubber bands and centrifuged at 750 × *g* force for 3 min. to drain the water from the wells.

6. The entire volume of the sequencing reaction was added to the centre of the Sephadex columns using a pipette and 25 μL of 0.1 mM EDTA was added to each well of the Sephadex plate.

7. The clean sequencing reaction was eluted by attaching a 96-well plate to the bottom of Sephadex plate, secure with rubber bands and centrifuged at 750 × *g* force for 3 min. The Sephadex plate was removed.

11. The 96-well collection plate was covered with a septum and placed into black plate bases and attached white plate retainer.

13. The assembled plate was stacked in ABI 3730xl capillary sequencer and the plate record was imported using Plate manager module of the Data Collection software (Applied Biosystems).

14. The sequencing run was started with Run Scheduler.

**(c) Protocols for extraction and PCR thermal conditions used at the Université Libre de Bruxelles**

Sequencing was realized at the Genoscope.

Total DNA was isolated with a NucleoSpin 96 plant kit (Macherey-Nagel), from 5 to 15 mg of silica-gel dried leave tissue. Polymerase chain reactions (PCRs) were carried out in a Biometra TProfessional Thermocycler.

PCRs for *mat*K and *rbc*L (total volume of 25 µL) included 1 µL of template DNA (10–100 ng),0.1 µL of Taq polymerase (Qiagen), 2.5 µL of PCR buffer, 1 µL of MgCl_2_ (25 mM), 0.5 µL of dNTP (10 µM), 0.25 µL of each primer (10 µM) and 18.9 µL of H_2_O. The cycling profile for PCR included an initial step of 3 min at 94 °C followed by 35 cycles of 20 s at 94 °C, 30 s at 52 °C and 1 min at 72 °C, followed by a final incubation at 72 °C for 10 min.

PCRs for *trn*H*-psb*A (total volume of 25 µL) included 1 µL of template DNA (10–100 ng), 0.1 µL of proofreading Phusion Taq (Finnzymes), 5 µL of PCR buffer, 1 µL of MgCl_2_ (25 mM), 0.5 µL of dNTP (10 µM), 0.25 µL of each primer (10 µM) and 16.9 µL of H_2_O.

The cycling profile for PCR included an initial step of 30 s at 98 °C followed by 35 cycles of 10 s at 98 °C, 20 s at 55 °C and 30 s at 72 °C, followed by a final incubation at 72 °C for 10 min.

Amplified fluorescent fragments were visualized using an ABI Prism 3730 automated DNA sequencer.

**(d) Protocol for sequencing used at the Genoscope**

Extraction and amplification were realized at the Université Libre de Bruxelles.

<http://www.genoscope.cns.fr/spip/>

**Purification of PCRs product with magnetic beads with AMPure XP® (Beckman Coulter, Inc.) 384 wells plate:**
Add 5µl PCR product to 9 µl AMPure XP
Incubate 5 min. at room T°
Put the plate 2 min. on a magnetic plate to separate beads from the solution and evacuate the cleared solution from the reaction plate.
2 washes with Ethanol 70%
Evaporation of Ethanol for 10 min. on the magnetic plate.

Out of the magnetic plate, add 15 µL elution buffer

Incubate at room T° for 2 min.
Put the plate 2 min. on a magnetic plate and transfer 15 µL of the solution in a new 384 wells plate.

**Sequencing reaction in 384-wells plate:**

1.5µL purified PCR product
BigDye® Terminator v3.1 Cycle Sequencing Kit :  0.25 µL
BigDye® Terminator v1.1/3.1 Sequencing Buffer (5X): 0.25 µL
Buffer Tris, MgCl_2_ (5X)  : 0.43 µL
Primer 5 µM : 1µL
H_2_O qsp 4 µL

Thermocycler program:

96°C   5 min
40 cycles:
96°C   15 sec
55°C   5 sec
60°C   4 min

4°C forever


Purification with Ethanol/Acetate and addition of 15 µl d'H_2_O

The model of the sequencer is 3730 XL   (Applied Biosystems).

**e) BLAST commands**

The BLAST method we used is implemented in Blasclust (version 2.2.23, ftp://ftp.ncbi.nih.gov/blast/executables/release). We used the default settings except W20 for *matK*. Here are the commands:

To format the database :

Formatdb –i database.fasta –pF –oF –aF –n namedatabase

To blast:

For *rbcL* and *trnH-psbA*:

megablast –FF –D3 –d namedatabase –i querysequences.fasta –o nameoutpufile

For *matK*:

megablast –FF –D3 –W20 –d namedatabase –i querysequences.fasta –o nameoutpufile
